# (In)frequently asked questions: On types of frequency and their role(s) in heritage language variability

**DOI:** 10.3389/fpsyg.2022.1002978

**Published:** 2022-11-23

**Authors:** Silvia Perez-Cortes, David Giancaspro

**Affiliations:** ^1^Department of World Languages and Cultures, Rutgers University–Camden, Camden, NJ, United States; ^2^Department of Latin American, Latino and Iberian Studies, University of Richmond, Richmond, VA, United States

**Keywords:** frequency, variability, heritage speakers, activation, lexicon

## Abstract

In recent years, researchers have become increasingly interested in exploring frequency as a source of variability in heritage speakers’ (HSs) knowledge of their heritage language (HL). While many of these studies acknowledge that frequency can affect the shape of HL grammars, there is still no clear consensus about (a) what “frequency” means in the context of HL acquisition and (b) how to operationalize its multiple subtypes. In this paper, we provide a critical overview of frequency effects in HL research and their relevance for understanding patterns of inter/intra-speaker variability. To do so, we outline how prior research has defined, measured, and tested frequency, and present—as well as evaluate—novel methodological approaches and innovations recently implemented in the study of frequency effects, including a new analysis of how self-reported lexical frequency reliably predicts HSs’ production of subjunctive mood in Spanish. Our aim is to highlight the immense potential of such work for addressing long-standing questions about HL grammars and to propose new lines of inquiry that will open up additional pathways for understanding HL variability.

## Introduction

Despite its exponential growth in recent years, the field of heritage bilingualism is still relatively young—especially in comparison to first (L1) and second (L2) acquisition research. While every year heritage language (HL) investigators continue to expand the reach of their work in terms of linguistic content and methodological approaches, research in this field has primarily focused on two lines of inquiry. The first one is centered around *between-group comparisons* and focuses on identifying areas where heritage speakers (HSs) differ from—or pattern like—other speaker groups, perhaps due to the enduring influence of L2 acquisition research—much of which examines differences between L2 learners and (monolingual) native speakers (e.g., White and Genesee, 1996). The second area of research involves *between-property comparisons*, which address the relative difficulty—or, to use a more commonly employed term, vulnerability (e.g., Polinsky and Scontras, 2020)—of different properties of the HL grammar (e.g., tense/aspect vs. mood morphology in the verbal domain: Montrul, 2009).

In our estimation, the pursuit of these two lines of inquiry has led to the vast majority of what we now know about HSs and their grammars. Nonetheless, we must recognize that, as informative as they have been, neither line of research addresses certain fundamental—and often overlooked—puzzles of HL research, especially those that involve the study of variability at the intraspeaker level. In this article, we advocate for increased attention to two promising yet less commonly investigated areas of study, each of which we will summarize here and then elaborate upon throughout the remainder of the paper. Critically, both approaches open the door to fine-grained analyses of frequency, a variable that has thus far received much more attention in L1 (e.g., Ambridge et al., 2015) and L2 (e.g., Ellis, 2002) research than in work with HSs, in spite of its evident potential in this area (e.g., O’Grady et al, 2011; Putnam and Sánchez, 2013; Montrul et al., 2014; Hur, 2020; López-Beltrán and Carlson, 2020; Giancaspro et al., 2022; Perez-Cortes, 2022b).

The first of the two categories of frequency that we will examine in this article, frequency of HL activation, involves the study of *between-speaker comparisons*, that is, the analysis of the unique grammatical systems that develop in the minds of individual HSs. Although inter-speaker heterogeneity is a defining—and well-recognized—characteristic of HSs (e.g., Montrul, 2016), we still have a lot to learn about the underlying factors that might cause two HSs with seemingly similar demographic/linguistic profiles to end up with what appear to be very differently shaped HL grammars. Why, for example, might one HS produce high rates of a certain inflection while another goes to great lengths to avoid it (e.g., Giancaspro, 2019)? Conceivably, part of this gap in our understanding results from the field’s longstanding reliance on group-level inferential analyses, which often take center stage in HL acquisition studies. Regardless of the reason for the scarcity of between-speaker analyses, variability at this level is a well-attested pattern that needs to be explored if we are to improve our understanding of the gradience of outcomes observed among HSs. After all, as much as we rely on group-level analyses in our quest to comprehend heritage bilingualism, linguistic systems form (and transform) in individual minds—not in groups. As such, our models and theories must also speak to the nature of these individual acquisitional paths.

The second type of frequency examined, which includes lexical frequency in its many instantiations, allows for *within-speaker comparisons*, that is, the study of variability that arises in individual HSs with a single HL property. When HSs differ from comparison groups, as they often do, contrasts tend to emerge in a variable rather than a categorical manner. For instance, for a given grammatical property, X, HSs will often produce both the instantiation that we usually see in control groups of monolingual speakers, as well as other (often innovative) variants (Flores, 2015). Despite its near ubiquity in HL research, this pattern of intra-speaker variability has also received relatively little attention from HL researchers, perhaps partially due to the complexity of accounting for such variability *via* formal linguistic theory, which often—though not exclusively (e.g., Sorace and Keller, 2005; Putnam et al., 2018)—views grammatical operations as categorical rather than gradient.

In the present paper, we argue for the importance of prioritizing the inclusion of frequency-based analyses in future empirical work, which enable the exploration of *between-speaker* and *within-speaker* comparisons. Despite their superficial differences, both types of comparisons have the potential to illuminate the multidimensional relationship between HSs’ linguistic knowledge, on one hand, and their language experience—operationalized via frequency—on the other. As we will outline in “Between-speaker comparisons: frequency of heritage language activation,” the analysis of HSs’ frequency of use/activation of their HL offers the unique chance for researchers to draw informative connections between speakers’ individual linguistic experience and their command of—or variability with—specific HL properties. We propose that by examining the extent to which individual HSs differ in their patterns of HL use, we can shed new light on HL heterogeneity, a puzzle that cannot be solved with *between-group* or *between-property* comparisons. Lexical frequency, which will be the focus of “Lexical frequency and its role in heritage grammars,” provides an opportunity for researchers to further interrogate HL variability, this time, at the level of individual speakers. Like between-speaker variability, intra-speaker variability, too, is a micro-level pattern that simply cannot be addressed by looking at the macro-level comparisons—specifically, between-speaker and between-property comparisons—that continue to predominate in the field. After reviewing different approaches to conceptualizing lexical frequency, we argue that subjective or self-reported lexical frequency—that is to say, HSs’ own evaluation of how often they hear/use certain words—can help us to explain individual HSs’ alternation between “target” and innovative variants of a given HL property. We conclude the paper in “Discussion and conclusion: Some final thoughts” by (a) proposing that frequency-based analyses should play a key role in our study of between-speaker and within-speaker HL research and (b) sketching out future directions for investigations that follow this blueprint.

## Between-speaker comparisons: frequency of heritage language activation

Regardless of theoretical background, HL researchers largely agree that HSs’ overall amount of experience with the HL, broadly conceived, will strongly affect the HL grammars that they ultimately develop (e.g., Kupisch and Rothman, 2018; Polinsky and Scontras, 2020; Montrul, 2021b *inter alia*). Thus, one would expect—all else equal—that a HS with extensive HL experience would perform in a less innovative (or more “target-like”) manner than a comparable peer whose use of the HL is relatively less frequent. Despite the consensus on this general—and perhaps obvious—point, researchers have dedicated relatively little attention to the question of how (and to what extent) between-speaker differences in HL usage/exposure/experience might lead different HSs to develop distinct patterns of grammatical knowledge with a given HL property. In fact, as noted by Daskalaki et al. (2019: 423), “no study to date has examined the differential effect of input quantity, as a continuous variable, within the same group of heritage speakers.” Before reviewing five studies that have shed light—directly or indirectly—on effects of HL usage/exposure/experience, we first summarize two papers that formalize how differences in HSs’ experience with the HL, sometimes referred to as frequency of activation, might lead to between-speaker differences in HL knowledge.

Putnam and Sánchez (2013), working from a generative theoretical framework, argue that HSs’ frequency of activation of their HL will impact the shape of their HL grammar. From this vantage point, HSs who use their HL less frequently might experience “a decline in the availability of FFs [functional features]” (p. 484) of their HL, which often manifests as innovative patterns of HL production and/or comprehension. Putnam and Sánchez’s conceptualization of heritage bilingualism creates a basic framework for accommodating differences in the performance of HSs. While those who frequently activate the HL—Stage 1 HSs in their terminology—are unlikely to exhibit major morphosyntactic innovations in their HL, HSs who use this language far less often (e.g., “Stage 3″ and “Stage 4” HSs) will demonstrate much more variability and innovation, in large part due to the increasing inaccessibility of FFs in their (relatively less activated) HL system. In a study of HSs’ production and comprehension of subjunctive mood, Perez-Cortes et al. (2019) provide evidence that is consistent with Putnam and Sánchez’s proposed stages. While high-activation HSs—operationalized as HSs with higher HL proficiency—performed in a more “target-like” manner with subjunctive mood, lower-activation HSs performed more variably, exhibiting increasingly prominent production/comprehension asymmetries as their proficiency in the HL decreased. More important than the specific details of their proposals, we believe, is these authors’ novel attempt to articulate how HSs with different levels of HL activation might end up with differentially innovative HL grammars.

### Measuring language activation and its effects on HL grammars: a complex enterprise

Since the publication of Putnam and Sánchez’s (2013) foundational work, additional evidence has emerged that aligns with its basic principles. A key example comes from studies that identify variability across HSs and descriptively explore the extent to which differences in exposure to the HL could be the source of said between-speaker contrasts. Cuza (2016), in a study of subject-verb (SV) inversion in the Spanish of younger and older child HSs, observed that younger HSs performed in a more “target-like” manner than their older counterparts, a finding that Cuza attributes—at least in part—to patterns of HL usage. Relative to the younger HSs, the older HSs in the study reportedly used less Spanish with their parents and siblings, which could, in principle, contribute to their differential knowledge of SV inversion. While suggestive, this trend could also have been caused by other differences between the two groups, such as older HSs’ emerging dominance in English.

Montrul and Sánchez-Walker (2013), in an extensive investigation of simultaneous and sequential HSs’ production of differential object marking (DOM) in Spanish, found that HSs produced less DOM in expected contexts than both monolingual and bilingual “baseline” groups. Far more revealing than these between-group differences, however, were the extensive between-speaker differences in the HS group—particularly in the case of the simultaneous HSs, whose production of DOM in expected contexts ranged from 0% to 100%. In an attempt to better understand these between-speaker differences, Montrul and Sánchez-Walker (2013) divided their HS participants into two groups—omitters, who produced DOM less than 80% of the time in expected contexts, and non-omitters—who produced DOM categorically. Post-hoc analyses revealed that, relative to the omitters, the non-omitters reported using Spanish more often in a variety of different situations, including with their parents, siblings, and friends. Though only a few of the differences between these two groups were statistically significant, these analyses point to the possibility that differences in HL usage can, in fact, trigger measurable between-speaker grammatical differences.

More recently, two additional studies have strengthened the claim that HSs’ frequency of experience with their HL shapes the variability of their HL grammatical systems. What sets these studies apart from Cuza (2016) and Montrul and Sánchez-Walker (2013) is that in each case, HL experience is seamlessly integrated into the inferential statistical modeling, thereby allowing for more reliable insight into the effects of this potentially critical explanatory variable. Dracos and Requena (2022) investigated child HSs’ production of a few different types of subjunctive mood morphology in Spanish, including, most relevantly for the present study, and volitional subjunctive forms (e.g., *quiero que bailes_SUBJ_* ‘I want you to dance’). Critically—and in contrast with previous studies of HSs and subjunctive mood—Dracos and Requena’s (2022) analyses incorporated information about HL usage/exposure, which they combined into a single variable that was included as a fixed factor in their mixed-effects statistical models. Results indicated that HSs with higher use of the HL (as reported by their caretakers) were significantly more likely to produce subjunctive mood morphology, pointing to HL experience as a factor in explaining certain between-speaker differences^1^. Complicating this finding, however, is the fact that HL proficiency—which is strongly correlated with HL use—was an even stronger predictor of HSs’ performance, thereby highlighting the difficulty of isolating HL experience as a cause of between-speaker differences.

Perhaps the most thorough attempts to connect the linguistic experiences of HSs to the grammatical systems they develop are López-Beltrán (2021), who tested the subjunctive mood knowledge of (adult) HSs of Spanish living in long-standing bilingual communities in New Mexico, and López Otero et al. (2021), who tested Spanish clitic production by adult HSs living in Brazil. Simplifying greatly, López-Beltrán found, using sophisticated statistical modeling, that HSs who reported higher use of Spanish were more sensitive to mood violations, as measured in a study of their pupil dilations, which reflect processing difficulty. Similarly, López Otero et al. (2021), who also explored effects of HL usage by treating it as a continuous variable in mixed effects models, found that HSs with higher HL usage were less likely to exhibit innovative clitic pronoun production.

### Moving forward: the future of studying frequency of HL use and exposure

In the studies reviewed above, we have seen preliminary evidence that between-speaker differences—that is to say, differences in the grammatical knowledge of different HSs—seem to be caused, at least in part, by differences in HSs’ frequency of use of and exposure to the HL. Weakening this conclusion, however, are two major methodological and epistemological concerns. First, it is not yet clear that language background questionnaires offer an accurate or reliable assessment of HSs’ actual patterns of HL use both (a) at the moment of data collection as well as (b) in earlier stages of their lives. This is especially concerning if HL use/exposure is more impactful during early childhood, as suggested by several researchers (e.g., Montrul, 2016; Silva-Corvalán, 2018; López-Beltrán, 2021). So how, exactly, have researchers attempted to quantify HSs’ frequency of HL use/exposure/experience? To illustrate the complexity of this task—and underline the need for new, methodologically-oriented work in this area—we briefly review the approaches employed in two of the studies presented in the previous section.

Dracos and Requena (2022) calculated (child) HSs’ experience with the HL by asking the HSs’ parents to provide approximate percentages of the time that their children hear/use the HL during the week, as well as on weekends. After receiving the responses, the researchers recoded the data into five different categories (0%–19%, 20%–39%, 40%–59%, 60%–79% and 80%–100%), which became levels of a fixed factor (HL usage/exposure) in their subsequent statistical modeling. While we applaud Dracos and Requena’s (2022) utilization of this variable in their analyses, we must acknowledge, too, the potential unreliability of the percentage estimates that they received. When a parent estimates the percentage of the time that their child uses the HL, what factors might they be considering (or not)? For example, how do they acknowledge, among other potential concerns, language mixing, asymmetric communication (e.g., parent speaks Spanish, and child responds in English), and the generally dynamic nature of HL use in a majority-language dominant society? The proportion of the HL that a child hears, for example, might vary greatly from week to week. In light of these challenges, it should not surprise us, perhaps, that HL proficiency—which may be a more direct measure of HL use/experience than questionnaire data—was a better predictor of subjunctive production than parental HL estimates^2^.

If it is challenging for HSs’ parents to estimate their children’s (current) HL use, it is likely even harder for adult HSs to accurately pinpoint the percentage of the time that they themselves used their HL (at the time of data collection or—cumulatively—throughout their lives). The adult HSs in López-Beltran’s (López-Beltrán, 2021) study, for example, were asked to determine the percentages of English and Spanish that they heard at home *before* beginning school, a period of time that likely predated their study participation by 13+ years. In households where parents exclusively used Spanish—and required their children to do the same—such estimates may, in fact, be quite reliable. (This may be why HL use was, after all, a statistically significant predictor in López-Beltran’s study). In households with more varied language practices, however, it is difficult to imagine that college-aged HSs could accurately and reliably recall percentages of their overall language usage during childhood. Consequently, differences in the middle of the (estimated) HL usage spectrum—e.g., between HSs who reported using their HL 60% of the time vs. those who reported using it 40% of the time—seem far less likely to effectively predict differences in speakers’ command of the HL in adulthood. Critically, this might be the case even if the “true” difference between 40% and 60% HL use does have important effects on adult HSs’ eventual grammatical knowledge.

A second (and related) concern with quantifying the effects of HL use, as mentioned in the preceding paragraphs, is that HL use/exposure is often strongly correlated with—and therefore hard to disentangle from—other potentially influential factors such as HL proficiency, age of acquisition of the majority language, and even formal education in the HL. As noted above, Dracos and Requena (2022)—as well as López-Beltrán—found that both HL use/experience *and* HL proficiency were statistically significant predictors of HSs’ grammatical performance, making it impossible to isolate the specific influence of HL use itself. (In the first of these studies, recall that proficiency was actually a stronger predictor than reported HL experience). Given this conceptual difficulty^3^, it may be the case—in spite of the suggestive evidence presented above—that our knowledge of how HL exposure/use affects HL grammars remains quite limited. Furthermore, even if we could design a perfectly reliable background questionnaire that allowed us to isolate the effects of HL usage from other potentially confounding variables, we would still face another major conceptual challenge. When differences in HL use lead different HSs to exhibit differential knowledge of a HL property, *where* exactly do these differences emerge?

To illustrate this conundrum, consider the following hypothetical. John and Carlos are both HSs of Spanish, though John reports using his HL two times as often as Carlos. (For the sake of argument, let us assume that John and Carlos are equivalent in terms of other pertinent background variables, thereby allowing us to isolate the effect of HL usage.) When John and Carlos complete an experimental task designed to assess their knowledge of mood morphology, John produces subjunctive in 80% of expected contexts while Carlos only does so in 40% of the same contexts. This hypothetical between-speaker difference would appear to indicate that Carlos’ HL usage affects his production of subjunctive mood. Nonetheless, this finding does not tell us where, at a fine-grained grammatical level, the two speakers differ from one another. It is possible, for example, that Carlos tends to use subjunctive with irregular verbs, or with forms that are more frequent, or even in contexts that are more likely to appear in academic/formal registers. In any case, the purpose of this example is to show that identifying between-speaker differences, though important, only provides indirect insight into individual patterns of HL development. To understand variability within a single speaker, that is to say, what factors lead Carlos to alternately produce both subjunctive, *and* indicative when subjunctive is expected, we will need to make within-speaker comparisons (e.g., with lexical frequency), which offer the micro-level perspective necessary for understanding individual HL grammatical patterns. In “Lexical frequency and its role in heritage grammars,” we elaborate on this point, using lexical frequency effects as an illustrative test case.

## Lexical frequency and its role in heritage grammars

In “Between-speaker comparisons: frequency of heritage language activation,” we discussed the advantages as well as limitations of how the field of HL acquisition has examined and modeled the development and outcomes of heritage bilinguals based on their patterns of language activation, that is, the frequency (or lack thereof) with which HSs use (and are exposed to) their HL. There is, however, another more fine-grained way in which frequency has been implemented to analyze patterns of language maintenance and change/innovation among heritage bilinguals. This second “type” of frequency (henceforth *lexical frequency*), which has recently emerged as an area of interest in HL research (Zyzik, 2016, 2019; Giancaspro, 2020; Gonzalez, 2020; Hur et al., 2020; Camacho, 2022; Giancaspro et al., 2022; Perez-Cortes, 2022b; *inter alia*), addresses the question of how the rate of occurrence of certain forms or structures in the HL input/output may affect their representation, processing, and use (Bybee, 1985, 2007).

While generative approaches to language acquisition have paid relatively little attention to the effects of lexical frequency (see Yang, 2004, 2015 for an exception), usage-based approaches, in contrast, have placed significant importance on this factor, arguing that “the structure and organization of a speaker’s linguistic knowledge is the product of language use or performance” (Diessel and Hilpert, 2016). From this perspective, increased (or decreased) exposure to a particular lexical item—based on its likelihood of appearing in the input—would affect how it is accessed, retrieved, and stored (Bybee, 1985, 2007; Poplack et al., 2013). Thus, highly frequent lexical items become the building blocks of grammatical categories, acting as exemplars around which related tokens cluster and establishing—and reinforcing—connections across multiple elements of language in what is known as entrenchment.

The nature and directionality of frequency effects appear to vary depending on the area of language under analysis. While high frequency collocations such as *I do not know* in English are especially vulnerable to phonological change or reduction (Bybee, 2006), high frequency morphological inflections, such as irregular past forms in English (i.e., *bought, went*), prove to be much more resistant to overregularization or simplification than less frequent counterparts (i.e., *snuck*, *dove*: Bybee, 1985). Since morphosyntax is the most commonly studied locus of variability in HL grammars (Putnam et al., 2022), and, furthermore, the data that we present in this paper comes from this domain, we narrow our focus in “Lexical frequency and its role in heritage grammars” to the effects of lexical frequency on HSs’ knowledge of morphosyntactic properties of the HL. To do so, we outline ways to operationalize lexical frequency, summarize cutting-edge studies and their proposals, and suggest future areas of research related to this topic.

As we will argue throughout this section, the limited exposure to (and use of) the HL often observed among HSs provides the perfect backdrop for the study of lexical frequency effects, in part, because this factor establishes a direct—and quantifiable—connection between speakers’ linguistic experience and how they represent and use language. It seems feasible, for example, that the relatively reduced input to which many HSs are exposed could drive them to rely more extensively on highly frequent HL items or structures, likely at the expense of lower frequency forms with which they have much less experience. As a result, properties or forms that are highly frequent in the (baseline) input might become more entrenched in the grammars of HSs, leading to lower levels of optionality in their use^4^. In contrast, less frequent HL forms would be more likely to favor the emergence of grammatical innovations (Backus, 2020) or morphosyntactic variability (Poplack et al., 2013; Perez-Cortes, 2022a). These hypotheses are compatible with recent theoretical proposals regarding the nature of the lexicon, especially those that advocate for an exoskeletal approach to morphology (Embick, 2015; Lohndal and Putnam, 2021). In particular, the adoption of a distributed view of lexical items (as the result of abstract morphosyntactic (*synsem*) features being mapped onto specific (morpho) phonological exponents) provides us with a systematic way to model and predict how frequency in the input could either reinforce such mappings, or allow for a disassociation between them, generating a wide range of outcomes that could have consequences at the level of production as well as representation (Perez-Cortes et al., 2019).

What makes considering the effects of lexical frequency most critical in future HL research, though, is that it allows us to account for differences that emerge at the individual level, that is, those that appear *within* speakers rather than *between* them. This change in perspective provides new and additional explanations to long-standing questions, such as why morphological variability tends to appear in certain forms but not in others, or how HSs’ lexical knowledge affects their overall linguistic development in the HL (Montrul and Mason, 2020; Montrul, 2021a).

### Operationalizing lexical frequency in research: the role of token, type, and lemma

Although it is common for acquisitional studies to refer to lexical frequency in broad terms, Ambridge et al. (2015), who work from a usage-based perspective, argue for a more specific use of this construct. With that in mind, what do HL researchers mean when they talk about the effects of lexical frequency? More often than not, observations about lexical frequency are centered around a word’s *token frequency*—i.e. its overall occurrence in the input^5^—as documented in large language corpora. According to Bybee (2007), forms that exhibit high token frequencies tend to 1) be more autonomous; and 2) have more lexical strength. Together, these factors make it more likely that speakers will access and retrieve frequent forms—which may be stored directly in their lexicon—as whole units or constructions, rather than assembling them derivationally (e.g., *walk* + −*ed* = *walked*). In the context of HSs, this would predict that more frequent items—from a token frequency perspective—would be, as a result of their autonomy/strength, more easily recognized and decoded in comprehension and less likely to exhibit variability in production^6^. Token frequency, however, is not the only existing category of lexical frequency, nor is it the only one that could generate predictions for HL acquisition. The construct of *type frequency*, likely the second most studied frequency category, captures the productivity of a particular pattern in language and accounts for analogical leveling in language acquisition, that is, the (over) application of a specific rule to forms that present relatively less common patterns (Hopper and Bybee, 2001).

Since token and type frequency interact with one another in complex ways (Bybee, 1995; Bybee and Thompson, 2000), we believe that researchers must—to the extent possible—carefully manipulate (or at least, control) these factors when conducting empirical analyses of HSs’ morphological knowledge. A perfect example of this can be found in the formation of past participles, tested among Spanish HSs by Mason (2019). As in English, Spanish past participles are classified based on whether their formation is considered regular or irregular. Regular participles are formed by adding the suffix *-(i)do/−(a) do* to the root of the verb, as in the case of *llegar*-*llegado* (‘to arrive/arrived’) or *ser-sido* (‘to be/been’). Irregular past participles, by contrast, can present a wider range of morphological instantiations, following patterns such as those in *hacer*-*hecho* (‘to do/done’), or *poner*/*puesto* (‘to put/put’). However, even within the subcategory of irregular past participles that end in-to, there are a number of different subpatterns, e.g., *romper/roto* (‘to break/broken’) or *escribir/escrito* (‘to write/written’), to give two quick examples. Thus, if we were to describe Spanish past participles based on their lexical frequency, we could do so in at least two different ways:

a) From a *token* frequency perspective, we could report and contrast their frequencies of occurrence in the input as documented by participant self-reports or by language corpora, such as the Davies’ Spanish NOW corpus (2012–2016) that we used to extract the information provided in Table 1. This would allow us to establish differences between how often specific verbal inflections (e.g., *sido*) are used relative to others (e.g., *hecho*). Though both *sido* and *llegado* are regular past participles, for example, *sido* is over five times more frequent; similarly, while *hecho* is an irregular participle, it is used about twice as often as the regular participle, *llegado*. These differences in token frequency—both within-and across-different types of regularity—could very well affect how HSs (and other Spanish speakers) learn and use participial forms.

**Table 1:** Token frequencies of regular and irregular Spanish past participles.

Regularity	Form	Token frequency (ranking/total participles)
Regular	Ser-sido (‘been’)	4,550,546 (1)
	Llegar-llegado (‘arrived’)	822,102 (14)
Irregular	Hacer—*hecho* (‘had’)	1,577,077 (2)
	Poner-puesto (‘put’)	463,155 (15)

b) From a *type* frequency perspective, we could analyze the productivity of the different word-formation patterns involved in the forms under consideration. As proposed by Mason (2019), in this particular case we would be able to identify two large clusters: those observed within regular participles such as *sido* and *llegado*, which present one of two different instantiations (*−ado* or-*ido*); and those observed in irregular forms (i.e., *hecho* and *puesto*). While the regular past participles-*ado* and-*ido* exhibit similarly high type frequencies, irregular participles have a wider range of different allomorphic subpatterns—Mason (2019:43) identifies up to seven—each of which may be relatively more or less common. Presumably, the differences in type frequency across irregular past participles, to give one example, could influence how HSs (and other Spanish speakers) develop their knowledge of participial forms.

Prior work on the acquisition of these structures among Spanish speakers (bilingual and monolingual) has found that irregular verbs (such as *hecho* or *puesto*) tend to be overregularized—e.g., to *hacido* or *ponido*–, especially during the first stages of acquisition (Clahsen et al., 2002; Soto-Corominas, 2021). In some cases, these “non-target-like” forms may even remain in the repertoire of adult bilinguals, especially if their exposure to Spanish is limited and/or they are not familiar with the specific verb where the suffix is featured (Montrul and Mason, 2020). Mason (2019) attributes this trend to differences in type frequency across past participles, whereby regular forms present more productive formation patterns (i.e., the use of-*ado/−ido*) than irregular forms. Pattern productivity alone, however, does not explain the gradience of outcomes observed in language acquisition, where overregularizations appear to be resolved in some verbs earlier than in others (i.e., *dicho* (‘said’) vs. *resuelto* (‘resolved’), as documented by Galaz et al. (2008). In this case, token frequency could help determine which particular irregular forms HSs might be more likely to regularize in an innovative way.

The effects of (type and/or token) frequency may also be examined by controlling their presence through careful study design and stimuli selection. This is precisely the strategy we adopted in our ongoing work on subjunctive mood among Spanish HSs in the US (see Giancaspro et al., 2022 for more information). The objective of this project was to revisit the study of a popular area of research among HSs of Spanish (i.e., subjunctive mood) by taking into account the effect of variables that had not been systematically controlled for in the past, such as the morphological regularity of the subjunctive forms tested, their type, and token frequency, and the modality of the proposition where the subjunctive forms were expected to appear. In contrast with previous research, we decided to control for the type frequency of the forms under analysis, limiting our selection of irregular verbs to those featuring a velar insert in their third person singular subjunctive inflections—instead of including forms with other types of irregularities, such as vocalic changes.

By controlling for type frequency, we were able to sidestep a key, potentially confounding variable and examine the effects of token frequency more directly, which gave rise to important differences across irregular verbs. The results in Table 2 indicate that while all high frequency irregular forms were similarly likely to elicit subjunctive mood from HSs, lower frequency irregular verbs elicited much more variability from HSs, as evidenced by both (a) HSs’ lower predicted probabilities of subjunctive production and (b) the wider confidence interval ranges for those predicted probabilities^7^. One clear exception to this pattern occurs in the case of *retenga* (‘to retain’), which, though infrequent, still elicits a very high rate of subjunctive production, a finding that opens up new areas of inquiry concerning word compositionality and the opacity/transparency of seemingly compound verbs. (Out of the irregular verbs tested, *tenga* is, by far, the most frequent, possibly making it easier for HSs to access and retrieve closely related subjunctive mood inflections such as *retenga*.) Overall, these findings not only provide additional insight on the effects of token frequency on subjunctive use, they also present a more nuanced description of morphological irregularity, which has generally been presented as a uniform, somewhat monolithic category.

**Table 2:** Predicted probability of subjunctive use as a function of token frequency.

			95% CI	
	Form elicited [*token frequency*^a^]	Predicted probability	Lower	Upper
More Frequent	Tenga [*712,671*]	0.96	0.90	0.99
	Salga [*119,654*]	0.97	0.93	0.99
	Ponga [*111,607*]	0.99	0.98	1.00
	Venga [*91,453*]	0.96	0.90	0.99
	Traiga [*13,048*]	0.98	0.96	0.99
Less frequent	Proponga [*11,192*]	0.93	0.83	0.97
	Convenga [*9,780*]	0.79	0.54	0.92
	Retenga [*1,912*]	0.97	0.91	0.98
	Extraiga [*1,345*]	0.93	0.84	0.97
	Sobresalga [*959*]	0.72	0.48	0.88

a Token frequencies were extracted from Davies’ NOW corpus (2012–2016).

Although less common, lexical frequency can also be examined from a lemmatic perspective, that is, considering the effects of all the inflectional variants of a particular form (verbal or nominal) all of which are represented with a single lemma^8^. The lemma cut, for instance, includes all possible forms of this verb, such as {*cutting, cut, cuts*…}, as well as the word’s nominal variants {*cut/cuts*}. Choosing to analyze the effects of verbal or nominal lexical frequency from a lemmatic perspective carries theoretical implications regarding how words are represented and accessed in the lexicon. In particular, it is assumed that the (cumulative) frequency of the paradigm will affect the lexical strength of individual—morphologically related—forms, making them more/less recognizable and likely to be retrieved. Adopting lemmatic frequency might be suitable for research where individual word differences are not central (i.e., measuring the extent to which the effects of (lemmatic) lexical frequency modulate the complexity of a text). Recent work dedicated to the study of frequency effects on morphological families, however, reports that the frequency of individual forms is more likely to predict variability in production, even if form similarity (between members of the same paradigm, for example) might play a role in how related items compete with each other (Bybee, 2002; Kapatsinski, 2010). These findings suggest that the adoption of lemmatic frequency might not be fitting if the focus of the study is on the development and acquisition of particular forms, where their individual token frequency—rather than the frequency of their complete inflectional paradigm—is relevant for the analysis. Let us imagine, for example, that we were interested in examining whether lexical frequency modulates the interpretation and use of different types of future (periphrastic vs. morphological) among US HSs of Spanish. In principle, frequency could be analyzed in two different ways: a) including information about the token frequencies of each type of future (i.e., *comprará* (‘(he/she) will buy’) [7936] vs. *va a comprar* (‘(he/she) will buy’) [3754]), or b) reporting the frequency of the complete paradigm in the form of lemmatic frequency (comprar [509875]). If we include the token frequency of all the forms involved, we would be able to explore whether (and how) the individual frequency of each inflection could affect HSs’ performance. The use of lemmatic frequency, in contrast, would limit our analysis to general frequency effects, allowing us to gauge the extent to which the frequency of a particular verb, regardless of its inflection, might drive HSs’ preference for one type of future over another. This broader perspective on frequency would allow us to capture verb-general effects, e.g., that Spanish speakers tend to use one type of future more with verbs that are collectively more frequent—that is to say, when all of its paradigms are collapsed together.

The previous discussion highlights the potential contributions of lexical frequency (in its different instantiations) to HL research, underscoring how individual speakers’ experience with a HL might shape their grammatical knowledge and use. In the next section, we provide a summary of recent investigations that have examined the effects of lexical frequency on HSs’ morphosyntactic development of the HL. After summarizing these studies, we present novel evidence that HSs’ subjective assessment of lexical frequency more effectively predicts their patterns of subjunctive mood production than corpus-based frequency metrics.

### Experimental approaches to lexical frequency effects in HL grammars

Putnam and Sánchez’s (2013) predictions regarding lexical frequency sparked a renewed interest in the study of how this variable might modulate HSs’ performance (Hur, 2020; Hur et al., 2020; Karayayla, 2021; López-Beltrán, 2021; Giancaspro et al., 2022; Perez-Cortes, 2022b, *inter alia*). As previously mentioned, the majority of the research in this area has focused on the domain of morphosyntax, with a particular emphasis on the acquisition of nominal and verbal inflection. Rather than manipulating it, some studies (Gor, 2019; López-Beltrán, 2021) have used token frequencies as a way to control HS participants’ familiarity with a particular selection of lexical items. Thus, instead of including verbs that are less frequent in the input, which are likely to have been less activated—and, as a result, more likely to exhibit increased variability–, these investigations only included highly frequent forms in the input, thereby giving participants the best chance to exhibit their HL knowledge. In the case of López-Beltrán’s (López-Beltrán, 2021) auditory pupillometry study, verb selection was made based on data compiled from the *Corpus Sociolingüístico de la Ciudad de México* (CSCM; Martín Butragueño and Lastra, 2011). Specifically, the researcher ensured that the frequency range of all subjunctive-triggering governors included in this receptive task (i.e., *Deseo que*, ‘I wish that’ or *Quiero que* ‘I want that’) was between 1 and 72 per 400,000 words. Additionally, the number of sentences that featured each governor was made proportional to its frequency, meaning that frequent triggers in the corpus appeared proportionally more often in the experimental task. Gor (2019) adopted a similar strategy in her study on the morphosyntactic knowledge of L2 learners and HSs of Russian. In particular, the investigator limited the vocabulary used in her grammaticality judgment task to words that appeared frequently in Russian language textbooks and that were also among the one thousand most frequent words in the Russian National Corpus.

The experimental designs adopted by Gor (2019) and López-Beltrán (2021) highlight the need to include stimuli that adequately represent the experience participants have with language. This is particularly relevant in the case of HSs, who may be more familiar with registers, styles or subsets of the lexicon that are not usually represented in traditional corpora. Karayayla (2021) addressed this particular question in her study of adult Turkish HSs’ use of inflectional suffix templates and the level of sophistication of the morphological forms they produce (when compared to Turkish monolinguals and recent immigrants). Based on previous work by Durrant (2013), Karayayla suggests that it is imperative to use frequency data that captures the characteristics of the input experienced by heritage bilinguals to reproduce as closely as possible their patterns of exposure. Accordingly, all type and lemmatic frequencies of the words and suffixes that appeared in her study were based on a corpus that included (informal) oral language that is spoken around UK-born HSs of Turkish. Information about the type frequency of the suffixes represented in the corpus was implemented to ensure that only those that were more productive would appear in the stimuli. Results from this study indicated that HSs exhibited lower nominal productivity than other groups, which translated into the application of nominal suffixes to a reduced—and primarily, high frequency—subset of Turkish nouns.

Lexical frequency, in particular token frequency, can also be manipulated to determine the extent to which it affects HSs’ ability to abstract grammatical knowledge from the input and generalize it across a wide range of lexical items. Perez-Cortes (2022b) sought to explore previously reported patterns of intraspeaker variability by focusing on the effects of token frequency on HSs’ preference and use of subjunctive in predicates that allow for variable mood selection. Participants in the study were a group of 35 intermediate-proficiency HSs of Spanish, who are among the most notoriously variable groups in HL research (Perez-Cortes et al., 2019). In two tasks (truth-value judgment and elicited production), Perez-Cortes tested two matrix verbs—*decir* (‘to say’) and *repetir* (‘to repeat’)—that represented both ends of the frequency spectrum, as seen in the contrasts illustrated in Table 3.

**Table 3:** Frequency values adapted from Perez-Cortes (2022: 158).

Matrix verb	Context	Token frequency (Davies NOW corpus)
Decir	Assertive (que + indicative)	76,962
	Jussive (que + subjunctive)	3,362
Repetir	Assertive (que + indicative)	237
	Jussive (que + subjunctive)	18

Results from a mixed-effects binary logistic regression indicated that Spanish HSs were more likely to interpret embedded clauses featuring subjunctive mood as commands, as would be expected in “baseline” Spanish, when the matrix verb introducing them was higher frequency (*M* = 0.65) rather than lower frequency (*M* = 0.51). Even though the type of matrix verb did not significantly affect HSs’ performance in a separate production task, a descriptive analysis of the data indicated that their probability of using subjunctive in jussive (indirect command) contexts was higher when the matrix verb was frequent (*M* = 0.64) than when it was not (*M* = 0.54). Token frequency has also been shown to affect intermediate Spanish HSs’ likelihood of using DOM in the expression of animate direct objects. In particular, Hur (2020) found that this group of bilinguals was more likely to favor the use of DOM with telic verbs that were more frequent (*M = *0.21), such as *cuidar* (‘to take care of’ [7531]) than with less frequent ones (*M = *0.07), such as *acariciar* (‘to pet’ [427]). Crucially, this pattern was not replicated among advanced-proficiency HSs, suggesting that as experience/proficiency with the HL grows, so does HSs’ ability to employ grammatical morphemes across a wider range of lexical items.

In line with the suggestions documented in Karayayla (2021), several studies have moved towards a more ecologically-valid approach of obtaining lexical frequency data, putting speakers’ individual experience with the HL at the forefront. (As Uygun & Clahsen (2021: 424) note, “frequencies for lexical entries may be highly variable for heritage speakers given their individual linguistic experience.”) Hur et al. (2020), in their investigation of the effects of token frequency on gender assignment and agreement in heritage Spanish, implemented a self-rating lexical frequency task (SRLFT)—adapted from López Otero (2020)—with this particular purpose in mind. In the SRLFT, HSs reported their use of and exposure to the 32 lexical items included in the subsequent elicited production and forced-choice tasks. Participants were asked how often they heard and used the items under examination using a 9-point Likert scale (1 = never, 2 = hardly ever, 3 = a few times a year, 4 = once a month, 5 = a few times a month, 6 = once a week, 7 = several times a week, 8 = once a day, 9 = several times a day), which resulted into a composite score for each lexical item that ranged from 2 to 18 (see Hur et al., 2020).

Results from a generalized linear mixed model including HSs’ responses across tasks revealed that lexical frequency—as measured by the SRLFT described above—facilitated gender assignment and agreement. In general, items that were deemed more frequent by participants favored the expected gender assignment and agreement, while those that were less frequently used and heard exhibited more variability.

The studies summarized thus far obtained (token) frequency information in two distinct ways: through language corpora or participant self-reports. To explore whether (and how) the adoption of these measures could affect how we conceptualize the effects of frequency in HSs’ performance, we reanalyzed data from Giancaspro et al. (2022)‘s study of Spanish HSs’ use of subjunctive mood in desiderative constructions (i.e., *Maria quiere que salgas pronto* ‘Maria wants you to leave [3psgSUBJ] early’). Using the Davies NOW Corpus (2012–2016), we collected the token frequency of all the subjunctive verbs used in our production task (*N =* 20), which included items along a wide frequency spectrum: from highly frequent forms (i.e., *tenga ‘*have’) to very infrequent ones (i.e., *sobresalga* ‘exceed’). Self-rated frequency was also examined using the results of a Lexical Experience Survey, which assessed participants’ (*N =* 42) use of and exposure to all experimental verbs using a four-point frequency scale where 1 meant that participants ‘never’ used a verb and 4 meant that they used that verb ‘very frequently’.

The data reported in Table 4 reveal a series of interesting observations. First, verbs at the low end of the (token) frequency spectrum based on their occurrence in the Davies’ NOW corpus, such as *parta* (‘split/cut’) [6,470] or *traiga* (‘bring’) [13,048] appeared to be rather frequent for HSs in both use (*parta:* 3.59/4; *traiga:* 3.91/4) and exposure (*parta:* 3.66/4; *traiga:* 3.92/4), perhaps because these forms may be more common in the household setting. Participants’ ratings also provided information about asymmetries in exposure and use that simply cannot be captured by traditional corpus data. Verbs like *proponga* (‘to propose’), *convenga* (‘to convene’) and *ceda* (‘to yield’) are good examples of this: in each case, HSs’ average exposure [range: 2.21–3.27] easily exceeds their self-reported use [range: 1.65–2.83].

**Table 4:** Corpus-based and self-rated token frequency (exposure and use) data.

Lexical item	Token frequency (Davies corpus)	Frequency of use (HS’ average out of 4)^a^	Frequency of exposure (HS’ average out of 4)
Tenga	712,671	3.85	3.85
Salga	119,654	3.98	4.00
Ponga	111,607	3.98	3.94
Venga	91,453	4.00	3.96
Traiga	13,048	3.91	3.92
Proponga	11,192	2.83	3.27
Convenga	9,780	1.67	2.21
Retenga	1,912	1.98	2.50
Extraiga	1,345	2.46	2.87
Sobresalga	959	2.30	2.67
Meta	21,597	3.76	3.80
Corra	10,993	3.63	3.71
Viva	27,637	3.93	3.88
Ceda	6,228	1.65	2.25
Parta	6,470	3.59	3.66
Prometa	1,366	3.30	3.46
Exceda	6,528	2.33	2.63
Comparta	56,158	3.78	3.85
Recorra	3,215	2.28	2.77
Sobreviva	3,800	2.91	3.25

a Since our study tested HSs’ knowledge and use of both indicative and subjunctive mood, participants’ self-ratings were based on the stimuli’s lemmas rather than their inflected indicative/subjunctive forms.

**Figure 1:**
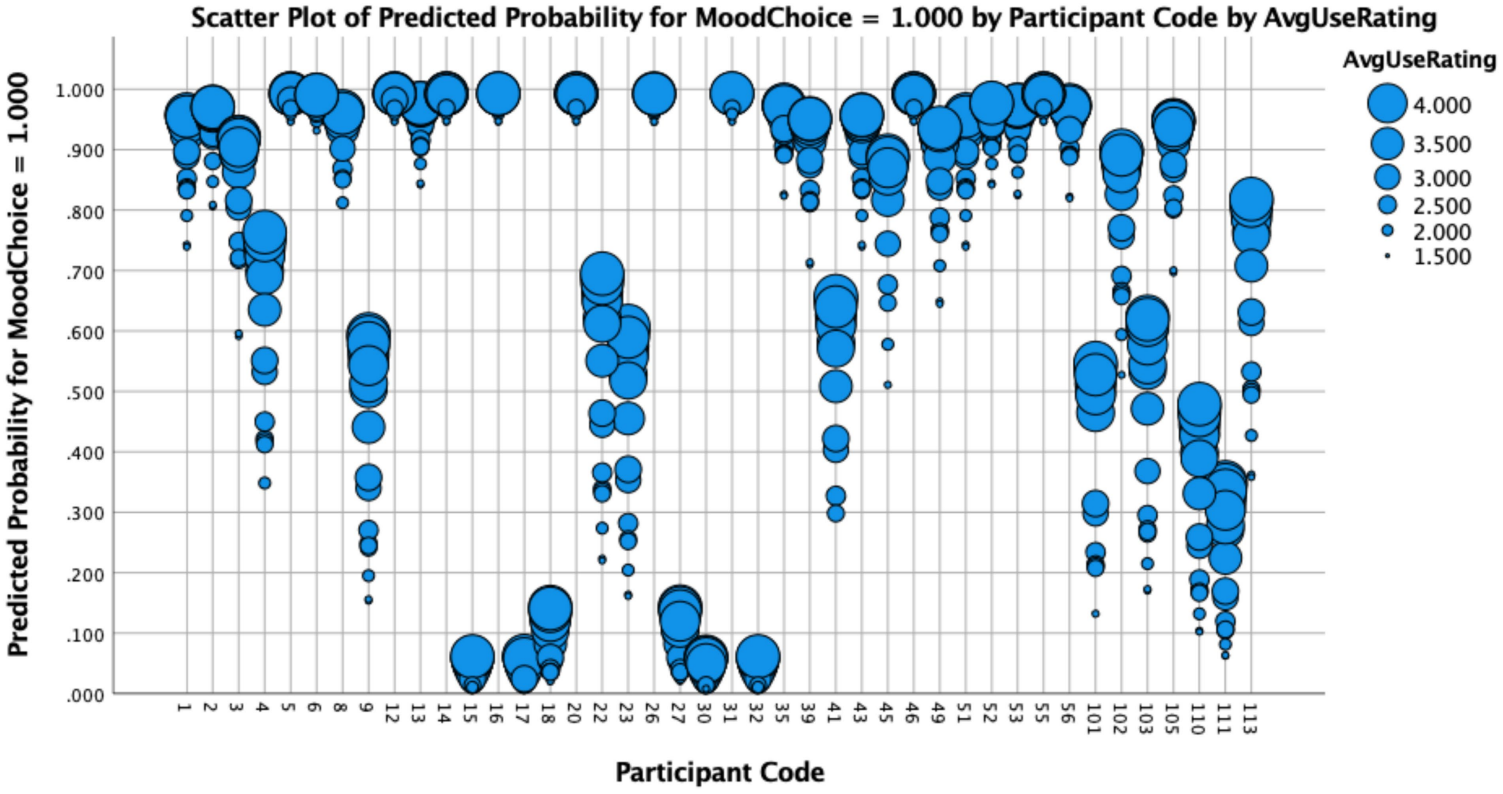
Individual participants’ predicted probability of subjunctive use as a function of self-reported average use.

Differences between these ways of capturing lexical frequency also emerged when we examined their statistical effects on HSs’ performance. To do so, we ran three separate binary logistic regression models—each with a different fixed factor (participants’ self-reported use (#1), exposure (#2) or items’ token frequency based on corpus data (#3))—and with subjunctive use, dummy-coded as 1 for subjunctive and 0 for indicative, as their dependent variable. In all cases, the best fitting models that converged included random slopes for Participant, as well as random intercepts for Item^9^. Results from these regressions revealed that while participants’ self-reported use (*ß* = 0.837, SE = 0.1538, *t* = 5.444, *p* < 0.001) and exposure (*ß* = 1.181, SE = 0.2004, *t* = 5.894, *p* < 0.001) were statistically significant predictors of their subjunctive use, token frequency based on the Davies’ corpus was not (*ß* = 0.0054, SE =0.3187, *t* = 1.739, *p* = 0.082). These findings suggest that relative to frequency metrics derived from large-scale corpora, self-reported frequency measures that reflect participants’ lived linguistic experience more accurately predict their likelihood of producing variability/grammatical innovations. Figure 1 depicts how participants’ self-reported use of the verbs in the study (Model #1) affected their production of subjunctive in expected subjunctive items^10^:

The results plotted in Figure 1 show that for most participants, the more frequently that they report using a lexical item, the more likely they are to produce it in the subjunctive (OR = 2.31; 95%, CI [1.70, 3.12], *p* < 0.001). As observed in the graph, verbs that participants reported using rarely (2) or never (1)—marked by smaller-sized blue circles—usually yielded the lowest predicted probabilities of subjunctive production. In contrast, verbs that participants reported using somewhat frequently (3) or very frequently (4)—marked by larger-sized blue circles—were more likely to elicit subjunctive mood inflections. Interestingly, participants whose performance was categorical at both ends of the probability scale—almost 40% of the sample—were not as affected by frequency as those who exhibited more variability, as in the case of Participant 9, whose verb-by-verb data we highlight in Table 5 below.

**Table 5:** Participant 9 (advanced HS) individual results as a function of reported use.

Verb	Self-reported use (out of 4)	Use of subjunctive (0 or 1)
Tener	4	1
Retener	3	1
Venir	4	1
Convenir	3	1
Traer	4	1
Extraer	**1**	**0 (indicative)**
Poner	4	1
Proponer	**2**	**0 (indicative)**
Salir	4	1
Sobresalir	**1**	**0 (indicative)**
Meter	3	0 (indicative)
Prometer	4	0 (indicative)
Ceder	**1**	**0 (indicative)**
Exceder	**2**	**0 (indicative)**
Correr	4	0 (indicative)
Recorrer	**2**	**0 (indicative)**
Partir	3	0 (indicative)
Compartir	4	0 (indicative)
Vivir	4	0 (indicative)
Sobrevivir	3	1

The bolded rows indicate verbs that participants reported using infrequently.

As indicated in Table 5, this participant, whose overall predicted probability of using subjunctive mood averaged 40%, did not produce subjunctive with any verb that they reported using either infrequently or “never”. In fact, 50% (6/12) of this participant’s innovative, indicative responses occurred with verbs that were relatively unfamiliar to them, and according to the self-rating task.

The information summarized thus far suggests that the study of lexical frequency—whether it is at the level of type, token, or lemma frequency—grants researchers the opportunity to tap into patterns of *intra-speaker variability*. However, despite the relevant role exerted by lexical frequency on HSs’ morphosyntactic development, we agree with Ambridge et al. (2015) that this factor alone cannot explain variability on its own. As these researchers note, lexical frequency—which in most cases is operationalized as the occurrence of individual tokens in the input—is likely to interact with other variables (i.e., regularity, phonological salience or semantic content) when modulating HL acquisition and maintenance, as reported in Giancaspro et al. (2022) and evident in Participant 9’s individual data. (Notably, Participant 9 uses much more subjunctive with irregular, as opposed to regular verbs.) The explanatory limitations of frequency, though, should not be seen as a disadvantage, especially given that potential interactions between frequency and other pertinent variables offer researchers multiple new avenues for better explaining HL variability.

## Discussion and conclusion: some final thoughts

The purpose of this article was twofold: first, we sought to clarify what is meant by ‘frequency effects’ in the field of HL acquisition research. To do so, we provided clear operationalizations of frequency both from a language activation lens, as well as from a lexical perspective. After laying out this critical groundwork, we then illustrated how further exploration of these frequency subtypes will help to illuminate two long-standing, yet relatively less studied patterns: (i) between-speaker variability, that is to say, differences in the linguistic knowledge of different HSs and (ii) within-speaker variability, meaning variability in individual HSs’ knowledge of particular HL forms (e.g., subjunctive mood). A second goal of the article was to serve as a point of departure for HL researchers who are interested in examining frequency—from either one (or both) of the perspectives mentioned—in their future studies. To this end, we presented a critical analysis of some of the field’s most relevant and recent work on frequency effects in the HL, paying particular attention to what should be considered best practices from theoretical as well as empirical vantage points. Among the most novel contributions of this overview, we believe, is the finding that self-reported lexical frequency—that is to say, HSs’ own subjective assessment of how frequently they hear/use certain words—appears to be a better predictor of their subjunctive mood variability than traditional, corpus-derived frequency metrics.

Before going any further, we believe that a couple of key clarifications are in order. First, while the present paper has prioritized the discussion of between-speaker and within-speaker comparative analyses, it is not our intention to dismiss the importance of more commonly studied contrasts—namely, between-group and between-property comparisons—in the study of heritage bilingualism. In fact, as we noted in the “Introduction,” the vast majority of the foundational work in our field has emerged from those two lines of inquiry, a reality which should not be overlooked. Our claim, instead, is that different comparative vantage points—including those that we have showcased in this paper—have different epistemological blind spots, meaning, essentially, that in order to appreciate the immense complexity of HL grammars, we must look at them from a more diverse variety of viewpoints. Just like between-group comparisons—e.g., comparing HSs to a baseline/control group—cannot shed light on why individual HSs might alternately produce two variants of a single form in a single HL context, within-speaker comparisons—like the analyses of lexical frequency effects presented in “Lexical frequency and its role in heritage grammars”—cannot explain why some HL properties (e.g., mood morphology) appear to be more “vulnerable” for HSs than others (e.g., tense/aspect morphology)^11^. Given the inherently complementary nature of between-group, within-speaker, and other perspectives on heritage bilingual knowledge, focusing (nearly) exclusively on one or two specific perspectives will necessarily lead to oversimplified understandings of HSs and the sophisticated linguistic systems that they develop and maintain. An even more concerning consequence of such epistemological uniformity, we believe, is that it could, if sufficiently conventionalized, make it increasingly difficult for researchers to even imagine other types of research questions whose answers might be needed in order to illuminate new paths forward for the field as a whole. Summarizing, then, it is our hope that the between-speaker and within-speaker comparisons that we promote in this paper both (a) complement, rather than replace, other types of comparisons, and (b) stimulate novel lines of inquiry, possibly (though not necessarily) related to the categories of frequency we discuss here.

While we recognize the enormous potential of the two varieties of frequency outlined in this paper, it is important to clarify, too, that neither is powerful enough to obviate other types of linguistic and non-linguistic explanations of HL grammars. In fact, as Ambridge et al. (2015) note, “a frequency effect can never be an explanation or answer in its own right” (p. 248), a point to which we will return later in this section. That said, if frequency is not—and cannot be—an explanation, why should researchers invest the time to address it carefully in their HL grammatical work? Do not we already have enough to worry about without diving into the frequency deep end?

One reason to embrace frequency is that frequency-effects—broadly conceived—appear to be an empirical reality of HL grammars. At the between-speaker level, differences in HSs’ frequency of experience with the HL seem to result in differences in the HL grammars that they ultimately develop. Recall, to recap an example from “Between-speaker comparisons: frequency of heritage language activation,” that the simultaneous HSs in Montrul and Sánchez-Walker (2013), who produced DOM in Spanish at rates ranging from 0% to 100%, were less likely to omit DOM if they used Spanish more frequently. Much more research is needed in this area—especially, work that builds patterns of HL use into statistical modeling—but the early returns, so to speak, certainly suggest that HSs who use the HL more often are more likely to develop generalized—rather than item-by-item—knowledge about HL grammatical properties, such as DOM or subjunctive mood. Relatedly, at the within-speaker level, it appears to be the case that HSs often develop “item-based” lexically-specific sensitivity to HL grammatical properties, that is, knowledge of certain morphemes/structures that only applies to specific subsets of the HL lexicon (e.g., gender with frequent nouns; mood with irregular verbs…etc.…) rather than to the HL lexicon in its entirety. To the extent that we can agree on the existence of these patterns—and the evidence, from our view, seems undeniable—posing (in)frequently asked questions about frequency in HL grammars is a necessary step in the field’s quest to understand HL grammatical systems as they are, and not just as they fit into our models.

There’s another reason to pursue frequency-based analyses in heritage bilingualism research. Though frequency is not, to reiterate, an explanation itself, investigating it and identifying some of its previously undiscovered effects can open the door to a number of novel analyses and research questions, many of which have the potential to reverberate far beyond HL research itself. As Ambridge et al. (2015) point out, when a so-called frequency effect is identified, it does not provide answers as much as it “poses a question: What type of learning mechanism is needed to yield the *particular type* of frequency effect observed?” Therefore, when Perez-Cortes (2022) documents token frequency effects on HSs’ interpretation and use of subjunctive mood or Mason (2019) finds that type frequency modulates HSs’ knowledge and use of present perfect and preterit forms, what might these specific patterns reveal about how HSs go about building (and maintaining) abstract grammatical knowledge? It is still very early, of course, but we suspect that facing—and then interrogating—these common HL patterns will challenge some of the binary conceptualizations that have thus far dominated not just HL acquisition research but also much of linguistic theory.

For reasons of space, we will conclude this paper by presenting two brief—and hopefully, inspirational—examples of how reflecting on—and taking into consideration—frequency effects, broadly defined, could deepen our understanding of HL grammatical complexity and actually improve existing explanations of widespread HL patterns and phenomena. A substantial proportion of research on HSs has focused on what they do not know and how they diverge from so-called baseline speakers (see Polinsky, 2018 or Montrul, 2016 for an overview). These between-group differences are undeniable, if not inevitable (Polinsky, 2016), yet, considering how little attention has been dedicated to controlling for (or manipulating) lexical frequency in experimental research on HSs, one wonders if the differences between-groups—which have formed the foundation of HL theories and models— may have been inadvertently inflated by the inclusion of infrequent (and/or high register) lexical items that are peripheral to HSs’ own linguistic life experiences^12^. Recent work, as highlighted in “Lexical frequency and its role in heritage grammars,” has started to address this oversight by considering lexical frequency when creating experimental items that are drawn from HS-specific corpora—and other sources—that more directly reflect participants’ linguistic experiences with the HL. A perfect example can be found in López-Beltrán’s innovative (López-Beltrán, 2021) study, where stimuli only consisted of highly frequent forms that were representative of HSs’ input in the HL. This methodological change had direct consequences in the results obtained, as HSs who participated in her tasks exhibited clearer sensitivity to subjunctive mood morphology than HSs in previously reported studies. This finding has the potential to serve as a methodological rebuke to so-called deficit perspectives on HL acquisition. If researchers test HSs on frequent items that form a key part of their HL experience, perhaps many of the HS vs. baseline differences will greatly diminish or even disappear altogether.

We have seen, thus far, that being more intentional about lexical frequency might help us to gain a more reliable representation of what HSs really know about their HL. On a similar note, we believe that lexical frequency might also help us to understand the nature of promising—yet still relatively underexplored—explanations of between-speaker differences, such as HL literacy/formal education. To illustrate this final point, let us reflect on Bayram et al’s (2017) work on passives in heritage Turkish. Summarizing briefly, Bayram et al. found that adolescent HSs were more likely to produce passive structures in their HL if they were more literate in Turkish. At one level, this finding constitutes an explanation of why some HSs appear to exhibit different knowledge than others. (This is a great example, in fact, of the type of between-speaker analysis that we hope to see more of in the field.) At another level, however, the finding that literacy drives between-speaker differences in passive production only raises another series of deeper, and perhaps more revealing questions, whose answers may be at least partially addressed by looking at lexical frequency. Are the more literate HSs in Bayram et al. (2017) more likely to use Turkish passives in general or only with higher register/lower frequency subsets of the Turkish lexicon, which they might be more likely to encounter in educational/written sources and settings? In asking this question, and we believe that other, similar questions can be asked of many other impactful studies in the field, we can better pinpoint the specific grammatical muscles that are strengthened by additional, formal HL experience, a finding that would have both theoretical and classroom implications.

In any case, we do not wish to belabor the point, but frequency-based analyses, in our view, raise interesting—even stimulating—questions and broaden our perspective of heritage grammars and their speakers. In a field as relatively young as HL acquisition research, pursuing new empirical questions and charting new methodological paths can only be a positive development, especially if those new directions, in acknowledging new layers of complexity, push us to more deeply reflect on the near ubiquitous (yet still understudied) patterns of between-speaker and within-speaker variability.

## Author contributions

All authors listed have made a substantial, direct, and intellectual contribution to the work and approved it for publication.

## Conflict of interest

The authors declare that the research was conducted in the absence of any commercial or financial relationships that could be construed as a potential conflict of interest.

## Publisher’s note

All claims expressed in this article are solely those of the authors and do not necessarily represent those of their affiliated organizations, or those of the publisher, the editors and the reviewers. Any product that may be evaluated in this article, or claim that may be made by its manufacturer, is not guaranteed or endorsed by the publisher.

## Footnotes

^1^For a similar finding with child HSs of Greek, see Daskalaki et al. (2019).

^2^Some studies, such as Unsworth (2013), have found that parental estimates of children’s language use/exposure are, in fact, effective predictors of children’s grammatical knowledge. The fact that different studies find differentially predictive effects of these estimates, however, may very well be attributable to (a) inconsistencies across the conceptualization of language use/exposure across different language background questionnaires (Kascelan et al., 2022) and/or (b) practical difficulties in disentangling language use/exposure from related and potentially confounding variables (e.g., proficiency).

^3^This challenge may also exist in the opposite direction, that is to say, in studies where another between-speaker variable (e.g., formal education) is identified as the underlying driver of between-speaker differences, but overall patterns of HL usage are not considered. Bayram et al. (2017), for example, report that HSs with higher literacy in Turkish are more likely to produce Turkish passive constructions than their less Turkish-literate counterparts, a finding that they attribute to literacy itself. (Notably, all HSs in the study produced passive forms, meaning that literacy cannot be a necessary precondition for their acquisition.) Since the authors do not present data about these groups’ overall usage of and exposure to Turkish, it is possible—especially if these variables are highly correlated with literacy—that their inclusion in the statistical modeling might have washed out some of the observed literacy effect.

^4^Consistent with this claim is a recent study by Torregrossa et al. (2022), which found that for HSs of European Portuguese living in Switzerland, HL experience factors (e.g., HL instruction) facilitated performance with more difficult (possibly, less frequent) cloze-test items in the HL (e.g., *ansiosa*: ‘anxious’) but not with lower difficulty (and, presumably, higher frequency) HL words/forms (e.g., *sol*: ‘sun’).

^5^Theoretically, one could (as seen in Giancaspro et al., 2022) account for token frequencies in the output (i.e., production) as well. Although work on monolingual acquisition has postulated that input/output frequency distributions tend to be extremely similar (DePaolis et al., 2011; Ambridge et al., 2015), this might not be the case for HSs, whose opportunities to productively use the HL are much more reduced than those of their monolingual peers (Montrul, 2016).

^6^While these tendencies have also been documented by Schmid and Köpke (2017) in the realm of L1 attrition, it is necessary to note—as pointed out by a reviewer—that they only hold in cases where variability is unintentional, that is, in situations where the speaker is not purposefully modifying a specific item (often prosodically) with a particular communicative intention in mind. See Kapatsinski et al. (2020) for a more detailed discussion.

^7^As noted by a reviewer, it is also possible that the width of the CI ranges was –in part– a result of log-odds mapping onto probabilities non-linearly, which could have contributed to the widening reported.

^8^Our operationalization of lemma differs from the one traditionally used in the production literature (i.e., Jescheniak and Levelt, 1994), aligning with the definition in the work of Gries (2009) and Knowles and Don (2004) instead).

^9^Best-fitting models were determined to be those with the lowest Akaike information criterion (AIC) values, in accordance with Heck et al. (2012). The first model (AIC = 3976.022), had an overall correct classification rate of 88.4%, the second one (AIC = 4243.107), had a classification rate of 88.3%, and the third one (AIC = 3884.784), with a classification rate of 86.9%.

^10^Despite finding several asymmetries in participants’ reported use and exposure to the verbs tested in our study, differences in how these two factors modulated their actual performance were minimal, hence our decision to only provide a graphic representation of one of them.

^11^For two mood-related examples of the between-property comparative vantage point needed for that type of question, see Montrul, 2009 and van Osch and Sleeman, 2018.

^12^This seems especially likely to happen, we believe, in cases where researchers’ own education in the target language far exceeds that of their HS participants. If we aren’t intentional in the selection of lexical items for our experiments—and we should note here that very few studies in HL verbal morphology comment on the verbs that are chosen for experimental tasks—it seems likely that lexical items that are infrequent (either in terms of their frequency in a corpus or, more importantly, from the perspective of individual HS participants themselves) will be oversampled in our tasks.

## References

[ref1] Ambridge B. Kidd E. Rowland C. F. Theakston A. L. (2015). The ubiquity of frequency effects in first language acquisition. J. Child Lang. 42, 239–273. doi: 10.1017/S030500091400049X, PMID: 25644408 PMC4531466

[ref2] Backus A. (2020). “Usage-based approaches” in The Routledge Handbook of Language contact. eds. Adamou E. Matras Y. (New York, NY: Routledge), 110–126. doi: 10.4324/9781351109154-8

[ref3] Bayram F. Rothman J. Iverson M. Kupisch T. Miller D. Puig-Mayenco E.. (2017). Differences in use without deficiencies in competence: passives in the Turkish and German of Turkish heritage speakers in Germany. Int. J. Biling. Educ. Biling. 22, 919–939. doi: 10.1080/13670050.2017.1324403

[ref4] Bybee J. L. (1985). Morphology: A study of the relation between meaning and form. Amsterdam: John Benjamins Publishing.

[ref5] Bybee J. (1995). Regular morphology and the lexicon. Lang. Cogn. Process. 10, 425–455. doi: 10.1080/01690969508407111

[ref6] Bybee J. (2002). Word frequency and context of use in the lexical diffusion of phonetically conditioned sound change. Lang. Var. Chang. 14, 261–290. doi: 10.1017/S0954394502143018

[ref7] Bybee J. (2006). From usage to grammar: the mind's response to repetition. Language 82, 711–733. doi: 10.1353/lan.2006.0186

[ref8] Bybee J. (2007). Frequency of Use and the Organization of Language. Oxford: Oxford University Press.

[ref9] Bybee J. Thompson S. (2000). Three frequency effects in syntax. Berkeley Linguistics Society 23, 378–388. doi: 10.3765/bls.v23i1.1293

[ref10] Camacho J. (2022). Paradigmatic uniformity: evidence from heritage speakers of Spanish. Languages 7:14. doi: 10.3390/languages7010014

[ref11] Caramazza A. (1997). How many levels of processing are there in lexical access? Cogn. Neuropsychol. 14, 177–208. doi: 10.1080/026432997381664

[ref12] Clahsen H. Aveledo F. Roca I. (2002). The development of regular and irregular verb inflection in Spanish child language. J. Child Lang. 29, 591–622. doi: 10.1017/s0305000902005172, PMID: 12109365

[ref13] Cuza A. (2016). The status of interrogative subject-verb inversion in Spanish-English bilingual children. Lingua 180, 124–138. doi: 10.1016/j.lingua.2016.04.007

[ref14] Daskalaki E. Chondrogianni V. Blom E. Argyri F. Paradis J. (2019). Input effects across domains: the case of Greek subjects in child heritage language. Second. Lang. Res. 35, 421–445. doi: 10.1177/0267658318787231

[ref15] Davies’ Spanish NOW corpus (2012–2016). Corpus of news on the web (NOW). Available at: https://www.corpusdelespanol.org/now/ (Accessed July 20, 2022).

[ref16] DePaolis R. A. Vihman M. M. Keren-Portnoy T. (2011). Do production patterns influence the processing of speech in prelinguistic infants? Infant Behav. Dev. 34, 590–601. doi: 10.1016/j.infbeh.2011.06.005, PMID: 21774986

[ref17] Diessel H. Hilpert M. (2016). Frequency effects in grammar. in *Oxford Research Encyclopedia of Linguistics*.

[ref18] Dracos M. Requena P. (2022). Child heritage speakers' acquisition of the Spanish subjunctive in volitional and adverbial clauses. Lang. Acquis., 1–28. doi: 10.1080/10489223.2022.207115635281590

[ref19] Durrant P. (2013). Formulaicity in an agglutinating language: the case of Turkish. Corpus Linguist. Linguist. Theory 9, 1–38. doi: 10.1515/cllt-2013-0009

[ref20] Ellis N. (2002). Frequency effects in language processing: a review with implications for theories of implicit and explicit language acquisition. Stud. Second. Lang. Acquis. 24, 143–188. doi: 10.1017/s0272263102002024

[ref21] Embick D. (2015). The Morpheme: A Theoretical Introduction. Berlin, Germany: Mouton de Gruyter.

[ref22] Flores C. (2015). Understanding heritage language acquisition: some contributions from the research on heritage speakers of European Portuguese. Lingua 164, 251–265. doi: 10.1016/j.lingua.2014.09.008

[ref23] Galaz X. Norambuena Muñoz C. Rivera Lazo M. (2008). Errores de sobrerregularización frecuentes en niños de entre tres y cinco años de edad. Cyber Humanitatis, 45. (Santiago, Chile: Universidad de Santiago de Chile).

[ref24] Giancaspro D. (2019). Over, under and around: Spanish heritage speakers' production (and avoidance) of subjunctive mood. Herit. Lang. J. 16, 44–70. doi: 10.46538/hlj.16.1.3

[ref300] Giancaspro D. (2020). “Not in the mood: Frequency effects in heritage speakers’ knowledge of subjunctive mood,” in Lost in Transmission: The Role of Attrition and Input in Heritage Language Development eds. B. Brehmer and J. Treffers-Daller (Amsterdam: John Benjamins), 72–97.

[ref25] Giancaspro D. Perez-Cortes S. Higdon J. (2022). (Ir) regular mood swings: lexical variability in heritage speakers’ oral production of subjunctive mood. Lang. Learn. 72, 456–496. doi: 10.1111/lang.12489

[ref26] Gonzalez B. H. (2020). The syntactic distribution of object experiencer psych verbs in heritage Spanish. Languages 5:63. doi: 10.3390/languages5040063

[ref27] Gor K. (2019). Morphosyntactic knowledge in late second language learners and heritage speakers of Russian. Herit. Lang. J. 16, 124–150. doi: 10.46538/hlj.16.2.2

[ref28] Gries S. T. (2009). What is corpus linguistics? Lang. Linguist. Compass 3, 1225–1241. doi: 10.1111/j.1749-818x.2009.00149.x

[ref29] Heck R. H. Thomas S. L. Tabata L. N. (2012). Multilevel and Longitudinal Modeling with IBM SPSS. New York, NY: Routledge.

[ref30] Hopper P. J. Bybee J. (2001). Frequency and the Emergence of Linguistic Structure. Amsterdam, Netherlands: John Benjamins Publishing.

[ref31] Hur E. (2020). “Verbal lexical frequency and DOM in heritage speakers of Spanish” in The Acquisition of Differential Object Marking. eds. Mardale A. Montrul S. (Amsterdam and Philadelphia: John Benjamins), 207–235.

[ref32] Hur E. Lopez Otero J. C. Sanchez L. (2020). Gender agreement and assignment in Spanish heritage speakers: does frequency matter? Languages 5:48. doi: 10.3390/languages5040048

[ref33] Jescheniak J. D. Levelt W. J. (1994). Word frequency effects in speech production: retrieval of syntactic information and of phonological form. J. Exp. Psychol. Learn. Mem. Cogn. 20, 824–843. doi: 10.1037/0278-7393.20.4.824

[ref34] Kapatsinski V. (2010). What is it I am writing? Lexical frequency effects in spelling Russian prefixes: uncertainty and competition in an apparently regular system. Corpus Linguist. Linguist. Theory 6, 157–215. doi: 10.1515/cllt.2010.007

[ref35] Kapatsinski V. Easterday S. Bybee J. (2020). Vowel reduction: a usage-based perspective. Rivista di Linguistica 32, 19–44. doi: 10.26346/1120-2726-146

[ref36] Karayayla T. (2021). A usage-based approach to productive use of inflectional patterns and level of lemma sophistication in adult heritage speakers’ performance: convergence on the immigrant variety. Linguist. Approaches Biling. 11, 753–782. doi: 10.1075/lab.18019.kar

[ref500] Kascelan D. Prévost P. Serratrice L. Tuller L. Unsworth S. De Cat C. (2022). A review of questionnaires quantifying bilingual experience in children: do they document the same constructs? Biling. Lang. Cogn. 25, 29–41. doi: 10.1017/S1366728921000390

[ref37] Knowles G. Don Z. M. (2004). The notion of a “lemma”: headwords, roots and lexical sets. Int. J. Corpus Linguist. 9, 69–81. doi: 10.1075/ijcl.9.1.04kno

[ref38] Kupisch T. Rothman J. (2018). Terminology matters! Why difference is not incompleteness and how early child bilinguals are heritage speakers. Int. J. Biling. 22, 564–582. doi: 10.1177/1367006916654355

[ref39] Lohndal T. Putnam M. T. (2021). The tale of two lexicon: decomposing complexity across a distributed lexicon. Herit. Lang. J. 18, 1–29.

[ref40] López Otero J. C. (2020). The acquisition of the syntactic and morphological properties of Spanish imperatives in heritage and second language speakers. Doctoral dissertation. New Brunswick, NJ: Rutgers the State University of new Jersey.

[ref41] López Otero J. C. Cuza A. Jiao J. (2021). Object clitic use and intuition in the Spanish of heritage speakers from Brazil. Second. Lang. Res.:026765832110176. doi: 10.1177/02676583211017603

[ref42] López-Beltrán P. (2021). Heritage speakers' online processing of the Spanish subjunctive: a comprehensive usage-based study. Doctoral dissertation. College Park, PA: Penn State University.

[ref43] López-Beltrán P. Carlson M. (2020). How usage-based approaches to language can contribute to a unified theory of heritage grammars. Linguist. Vanguard 6. doi: 10.1515/lingvan-2019-0072

[ref44] Martín Butragueño P. Lastra Y. (2011). Corpus sociolingüístico de la ciudad de México. México: El Colegio de México.

[ref45] Mason S. A. (2019). The influence of task factors and language background on morphological processing in Spanish. Doctoral dissertation. Urbana-Champaign, IL: University of Illinois at Urbana-Champaign.

[ref46] Montrul S. (2009). Knowledge of tense-aspect and mood in Spanish heritage speakers. Int. J. Biling. 13, 239–269. doi: 10.1177/1367006909339816

[ref47] Montrul S. (2016). The acquisition of heritage languages. Cambridge, United Kingdom: Cambridge University Press.

[ref48] Montrul S. (2021a). “Morphology in Spanish heritage language grammars” in The Routledge Handbook of Spanish Morphology. eds. Fábregas A. Acedo-Matellán V. Armstrong G. Cuervo M. C. Payet I. P. (New York, NY: Routledge), 538–549.

[ref49] Montrul S. (2021b). Representational and computational changes in heritage language grammars. Herit. Lang. J. 18, 1–30. doi: 10.1163/15507076-12340011

[ref50] Montrul S. Davidson J. De La Fuente I. Foote R. (2014). Early language experience facilitates the processing of gender agreement in Spanish heritage speakers. Biling. Lang. Congn. 17, 118–138. doi: 10.1017/s1366728913000114

[ref51] Montrul S. Mason S. A. (2020). Smaller vocabularies lead to morphological overregularization in heritage language grammars. Biling. Lang. Congn. 23, 35–36. doi: 10.1017/s1366728919000427

[ref52] Montrul S. Sánchez-Walker N. (2013). Differential object marking in child and adult Spanish heritage speakers. Lang. Acquis. 20, 109–132. doi: 10.1080/10489223.2013.766741

[ref53] O'Grady W. Kwak H. Y. Lee O. Lee M. (2011). An emergentist perspective on heritage language acquisition. Stud. Second. Lang. Acquis. 33, 223–245. doi: 10.1017/S0272263110000744

[ref54] Perez-Cortes S. (2022a). On complexity and divergence in heritage language grammars: the case of double mood selection in reported speech contexts. Stud. Second. Lang. Acquis. 44, 818–842. doi: 10.1017/s0272263121000589

[ref55] Perez-Cortes S. (2022b). Lexical frequency and morphological regularity as sources of heritage speaker variability in the acquisition of mood. Second. Lang. Res. 38, 149–171. doi: 10.1177/0267658320918620

[ref56] Perez-Cortes S. Putnam M. T. Sánchez L. (2019). Differential access: asymmetries in accessing features and building representations in heritage language grammars. Languages 4:81. doi: 10.3390/languages4040081

[ref58] Polinsky M. (2016). Structure vs. use in heritage language. Linguist. Vanguard 2. doi: 10.155/lingvan-2015-0036

[ref400] Polinsky M. (2018). Heritage languages and their speakers (Cambridge, United Kingdom: Cambridge University Press).

[ref59] Polinsky M. Scontras G. (2020). Understanding heritage languages. Biling. Lang. Congn. 23, 4–20. doi: 10.1017/s1366728919000245

[ref60] Poplack S. Lealess A. Dion N. (2013). The evolving grammar of the French subjunctive. Int. J. Latin Roman. Linguist. 25, 139–195. doi: 10.1515/probus-2013-0005

[ref61] Putnam M. Carlson M. Reitter D. (2018). Integrated, not isolated: definining typological proximity in an integrated multilingual architecture. Front. Psychol. 8:2212. doi: 10.3389/fpsyg.2017.02212, PMID: 29354079 PMC5758582

[ref62] Putnam M. Sánchez L. (2013). What's so incomplete about incomplete acquisition? A prolegomenon to modeling heritage language grammars. Linguist. Approaches Biling. 3, 478–508. doi: 10.1075/lab.3.4.04put

[ref63] Putnam M. Schwarz L. Hoffman A. D. (2022). “Morphology of heritage languages” in The Cambridge Handbook of Heritage Languages and Linguistics. eds. Montrul S. Polinsky M. (Cambridge: Cambridge University Press), 613–643.

[ref64] Schmid M. S. Köpke B. (2017). The relevance of first language attrition to theories of bilingual development. Linguist. Approaches Biling. 7, 637–667. doi: 10.1075/lab.17058.sch

[ref65] Silva-Corvalán C. (2018). Simultaneous bilingualism: early developments, incomplete later outcomes? Int. J. Biling. 22, 497–512. doi: 10.1177/1367006916652061

[ref66] Sorace A. Keller F. (2005). Gradience in linguistic data. Lingua 115, 1497–1524. doi: 10.1016/j.lingua.2004.07.002

[ref67] Soto-Corominas A. (2021). “Morphology and L1 acquisition” in The Routledge Handbook of Spanish Morphology. eds. Fábregas A. Matellán V. A. Armstrong G. Cuervo M. C. Payet I. P. (New York, NY: Routledge), 513–525.

[ref68] Torregrossa J. Flores C. Rinke E. (2022). What modulates the acquisition of difficult structures in a heritage language? A study of Portuguese in contact with French, German, and Italian. Biling, 1–14. doi: 10.1017/S1366728922000438

[ref600] Unsworth S. (2013). Assessing the role of current and cumulative exposure in simultaneous bilingual acquisition: The case of Dutch gender. Biling. Lang. Cogn. 16, 86–110. doi: 10.1017/S1366728912000284

[ref69] Uygun S. Clahsen H. (2021). Morphological processing in heritage speakers: a masked priming study on the Turkish aorist. Biling. Lang. Congn. 24, 415–426. doi: 10.1017/S1366728920000577

[ref70] van Osch B. Sleeman P. (2018). Spanish heritage speakers in the Netherlands: linguistic patterns in the judgment and production of mood. Int. J. Biling. 22, 513–529. doi: 10.1177/1367006916654365

[ref71] White L. Genesee F. (1996). How native is near-native? The issue of ultimate attainment in adult second language acquisition. Second. Lang. Res. 12, 233–265. doi: 10.1177/026765839601200301

[ref72] Yang C. (2004). Universal grammar, statistics or both? Trends Cogn. Sci. 8, 451–456. doi: 10.1016/j.tics.2004.08.006, PMID: 15450509

[ref73] Yang C. (2015). For and against frequencies. J. Child Lang. 42, 287–293. doi: 10.1017/s0305000914000683 25644412

[ref74] Zyzik E. (2016). “Toward a prototype model of the heritage language learner,” in Innovative Strategies for Heritage Language Teaching: A Practical Guide for the Classroom. eds. Fairclough M. Beaudrie S. (Washington, DC: Georgetown University Press), 19–38.

[ref75] Zyzik E. (2019). Incomplete acquisition from a usage-based perspective: a response to Domínguez, Hicks, and Slabakova. Stud. Second. Lang. Acquis. 41, 279–282. doi: 10.1017/s0272263119000330

